# Systematic analysis of cuproptosis abnormalities and functional significance in cancer

**DOI:** 10.1371/journal.pone.0300626

**Published:** 2024-04-04

**Authors:** Shang Rumin, Xiangming Han, Cui Zeng, Fei Lv, Rong Fang, Rongrong Gong, Xiaochang Tian, Xiangwu Ding

**Affiliations:** 1 Department of Gastroenterology, Wuhan Pu’ai Hospital, Tongji Medical College, Huazhong University of Science and Technology, Wuhan, 430000, China; 2 Department of Oncology, Nanjing Drum Tower Hospital, Nanjing, 210008, China; 3 Department of Gastroenterology, Wuhan Fourth Hospital, Wuhan, 430000, China; McGill University, CANADA

## Abstract

**Background:**

Cuproptosis is a recently discovered type of cell death, but the role and behavior of cuproptosis-related genes (CuRGs) in cancers remain unclear. This paper aims to address these issues by analyzing the multi-omics characteristics of cancer-related genes (CuRGs) across various types of cancer.

**Method:**

To investigate the impact of somatic copy number alterations (SCNA) and DNA methylation on CRG expression, we will analyze the correlation between these factors. We developed a cuproptosis index (CPI) model to measure the level of cuproptosis and investigate its functional roles. Using this model, we assessed the clinical prognosis of colorectal cancer patients and analyzed genetic changes and immune infiltration features in different CPI levels.

**Results:**

The study’s findings indicate that the majority of cancer-related genes (CuRGs) were suppressed in tumors and had a positive correlation with somatic copy number alterations (SCNA), while having a negative correlation with DNA methylation. This suggests that both SCNA and DNA methylation have an impact on the expression of CuRGs. The CPI model is a reliable predictor of survival outcomes in patients with colorectal cancer and can serve as an independent prognostic factor. Patients with a higher CPI have a worse prognosis. We conducted a deeper analysis of the genetic alterations and immune infiltration patterns in both CPI positive and negative groups. Our findings revealed significant differences, indicating that CuRGs may play a crucial role in tumor immunity mechanisms. Additionally, we have noticed a positive correlation between CuRGs and various crucial pathways that are linked to the occurrence, progression, and metastasis of tumors.

**Conclusions:**

Overall, our study systematically analyzes cuproptosis and its regulatory genes, emphasizing the potential of using cuproptosis as a basis for cancer therapy.

## 1 Introduction

The study of the was a novel cell death type of cuproptosis [1] first carried out by Tsvetkov et al. (2022), which differs from known regulated cell death types, including necroptosis, pyroptosis, ferroptosis, parthanatos, entosis, NETosis and lysosome-dependent cell death (LCD). Cuproptosis is primarily caused by the combination of copper ions with the ferredoxin 1-mediated protein lipoylation process in the pyruvate dehydrogenase (PDH) complex, which is a component of the tricarboxylic acid cycle. The accumulation of mitochondrial proteins that are bound to copper and lipoylated, along with the resulting depletion of Fe-S cluster protein, induces proteotoxic stress which ultimately leads to a distinct form of cell death.

Tsvetkov and colleagues have identified 19 genes that are involved in cuproptosis, a process of cell death caused by copper overload. These genes include NFE2L2, NLRP3, ATP7B, ATP7A, SLC31A1, FDX1, LIAS, LIPT1, LIPT2, DLD, DLAT, PDHA1, PDHB, MTF1, GLS, CDKN2A, DBT, GCSH, DLST and play important roles in regulating this process. Among these genes are necessary ones for cuproptosis such as LIAS, DLD, DLAT, FDX1, and LIPT1; SLC31A is a gene that promotes cuproptosis while ATP7B and ATP7A inhibit it. Tsvetkov et al. also conducted a CRISPR-Cas9 screening and found that MFTI, GLS, and CDKN2A inhibit cuproptosis while PDHA1 and PDHB promote it. Apart from the aforementioned genetic factors, glutathione (GSH) can also function as a cuproptosis inhibitor.

The study of genes related to cuproptosis has revealed the complex biological functions associated with it. Several studies have examined the effects of LIPT1 on the proliferation, invasion, and migration of HCC (hepatocellular carcinoma) cells. Therefore, targeting LIPT1 could be a promising treatment approach for HCC [2]. Yang et al. related to cuproptosis is significantly enriched in Treg cells and macrophages, and closely associated with the exhaustion of proliferative T cells, providing a basis for combined immunotherapy for HCC [3]. Xu et al. developed DOX@Fe/CuTH, which can promote the production of H_2_0_2_ and consumption of GSH to amplify cellular oxidative stress and synergistic cuproptosis, effectively inhibiting tumor growth [4]. Collectively, these studies outline a critical role for cuproptosis in cancer progression and treatment. Despite the known association of copper ions with various cancers, including breast cancer [5] first carried, gliomas [6], and renal clear cell carcinoma [7], there is currently a lack of understanding regarding the expression and variation patterns of CuRGs in different types of cancer. Conducting a comprehensive investigation on the dysregulation of cuproptosis across various types of cancers would be beneficial.

Currently, colorectal cancer (CRC) is one of the most common malignant tumors with an incidence rate of 10% and a mortality rate of 9.4%, ranking third and second globally, respectively [8,9]. The prognosis of CRC is closely related to the immune microenvironment [10], cell apoptosis [11], ferroptosis [12], and tumor mutation burden [13,14]. Previous studies have shown that copper has an anti-proliferative effect on CRC [15]. However, there is no research that confirms whether cuproptosis has diagnostic and prognostic value in colorectal cancer. Therefore, it is necessary to further study the association between the prognosis of colorectal cancer and cuproptosis to provide potential biomarkers and targets for the diagnosis and treatment of colorectal cancer.

In the current study, we performed a comprehensive analysis of genomic variations and expression profiles of CuRGs across 20 distinct cancer types. The most important clinically relevant finding was cuproptosis is closely related to the survival of colorectal cancer, renal clear cell carcinoma and endometrial cancer. The immune microenvironment and cancer hallmarks were found to be associated with cuproptosis. Furthermore, the study confirmed that the CPI is a standalone independent factor for colorectal cancer. The results emphasize the crucial contribution of cuproptosis to cancer and offers insights into the molecular mechanisms associated with cuproptosis and potential therapeutic development.

## 2 Methods and materials

### 2.1 Collection of pan-cancer datasets

The mRNA-Seq expression data and mutation data of 20 types of cancers (Table 1), including (BLCA, BRCA, CESC, CHOL, COAD, ESCA, GBM, HNSC, KICH, KIRC, KIRP, LIHC, LUAD, LUSC, PAAD, PRAD, READ, STAD, THCA, UCEC), were downloaded from the PanCanAtlas Publications (https://gdc.cancer.gov/about-data/publications/pancanatlas). The missing values in the data were imputed using the nearest neighbor algorithm in the R package "impute" to increase the coverage of genes. Copy number alteration thresholded data, masked copy number segmentation data, and DNA methylation 450K data of those twenty cancers, were all downloaded from Firehose (http://gdac.broadinstitute.org). We obtained clinical information, including tumor staging, TNM staging, and survival prognosis data for different types of cancers from the PanCanAtlas Publications.

**Table 1:** Tumor full name and abbreviation.

Cancer Abbreviation	Cancer Name
BLCA	Bladder Urothelial Carcinoma
BRCA	Breast invasive carcinoma
CESC	Cervical squamous cell carcinoma and endocervical adenocarcinoma
CHOL	Cholangiocarcinoma
COAD	Colon adenocarcinoma
ESCA	Esophageal carcinoma
GBM	Glioblastoma multiforme
HNSC	Head and Neck squamous cell carcinoma
KICH	Kidney Chromophobe
KIRC	Kidney renal clear cell carcinoma
KIRP	Kidney renal papillary cell carcinoma
LIHC	Liver hepatocellular carcinoma
LUAD	Lung adenocarcinoma
LUSC	Lung squamous cell carcinoma
PAAD	Pancreatic adenocarcinoma
PRAD	Prostate adenocarcinoma
READ	Rectum adenocarcinoma
STAD	Stomach adenocarcinoma
THCA	Thyroid carcinoma
UCEC	Uterine Corpus Endometrial Carcinoma

### 2.2 Analysis of differential mRNA expression

To identify genes that are differentially expressed between tumor and normal tissue, differential analysis was performed using the DESeq2 package, where the absolute value of fold change>1.5 and adjusted *P*-value < 0.05 were considered as differential expression genes (DEGs). We calculated the number of DEGs of CuRGs for each type of cancer.

### 2.3 Analysis of somatic copy number alterations (SCNAs)

To assess the copy number alteration of each gene, we considered heterozygosity and homozygosity of amplification and deletion. SCNAs at the gene level were calculated using the Gist2 software, representing the q-value of abnormal regions of SCNAs. The samples corresponding to each CuRGs were divided into amplification and deletion groups using thresholds of SCNA > 0.05 and SCNA < -0.05, respectively. The amplification rate and deletion rate of CuRGs were calculated as follows: amplification rate = number of amplified samples/total number of samples, deletion rate = number of deleted samples/total number of samples, where the total number of samples = number of amplified samples + number of deleted samples. To determine the correlation between SCNAs and the expression of CuRGs, we calculated the Pearson correlation coefficient. If the resulting *P*-value was less than 0.05, we considered it a significant association between SCNA and gene expression level.

### 2.4 DNA methylation analysis

We utilized the methylation 450k dataset from ChAMP, a built-in probe feature in R package, to annotate gene promoter methylation probes (TSS1500, TSS200). To identify differentially methylated genes with hypomethylated or hypermethylated in each type of cancer, we conducted Wilcoxon rank-sum tests between the tumor and normal groups for methylation of each gene with a *P*-value cutoff of 0.05. To assess correlation between transcriptional expression of CuRGs and promoter DNA methylation Beta values, we calculated Pearson’s correlation and considered it significant if the *P*-value was less than 0.05.

### 2.5 Constructing a Cuproptosis Index (CPI) model

Based on the gene set scoring method proposed by Sotiriou et al. using gene expression levels, the Gene Expression Grade Index (GGI), we also established a score called CPI to summarize the similarity between the expression profile and the level of cuproptosis. For this analysis, we utilized the combined data from both COAD (colon adenocarcinoma) and READ (rectum adenocarcinoma) datasets. The genes involved in constructing the model were divided into positive components: CDKN2A, GCSH, LIPT2, and negative components: ATP7A, ATP7B, DBT, DLAT, DLD, DLST, FDX1, GLS, LIAS, LIPT1, MTF1, NFE2L2, NLRP3, PDHA1, PDHB, SLC31A1. The enrichment score (ES) of the positive and negative gene sets were calculated using single-sample gene set enrichment analysis (ssGSEA) in the R package GSVA, and the normalized difference between the ES of the positive and negative components was defined as the CPI to calculate the level and trend of cuproptosis in analyzed tissue samples. Wilcoxon rank-sum test was used to perform differential analysis on the CPI indices of the tumor and normal groups in each type of cancer. *P*-value < 0.05 indicated a significant difference between the two groups.

### 2.6 Univariate Cox regression analysis of CuRGs and CPI survival analysis

To evaluate which genes in CuRGs played an important role in prognosis, a univariate Cox regression model was established for each CRG. A gene was considered as a protector if HR < 1 and *P* < 0.05, and a gene was considered as a risk factor if HR > 1 and *P* < 0.05. Compared with survival analysis using a single gene, we used the CPI, which measures the overall level and trend of cuproptosis, to perform survival analysis. The tumor patients were divided into positive and negative groups based on the positive and negative CPI values, and the survival curves for 20 types of cancers were plotted and log-rank test was performed. P < 0.05 indicated a significant difference in the survival time between the two groups. Specially, in the TCGA-COAD dataset, out of 463 available samples, only 286 with complete survival data were included in our analysis. This was due to the exclusion of samples lacking crucial survival information. Conversely, all 91 samples in the TCGA-READ dataset, which had complete survival data, were utilized. Both datasets were confined to adenocarcinomas only.

### 2.7 Independent prognostic analysis of CPI in colorectal cancer

CPI was evaluated for its ability to reflect the prognosis of patients independently of other clinical factors, including age, gender, pathological grade, and pathological T and N staging. A multiple-factor Cox regression model was established using the "rms" and "survival" R packages. Next, a prognostic nomogram was established based on the independent prognostic factors. A calibration curve was also constructed to evaluate the performance of the model based on the above indicators.

### 2.8 Genomic alteration analysis of CRC based on CPI grouping

We created independent waterfall plots for the top 20 genes with the highest mutation frequency in CPI-positive and CPI-negative groups. Additionally, we conducted GO and KEGG pathway analyses separately to determine which molecular pathways were impacted by these highly variable genes in each group. In calculating the tumor mutation burden (TMB), we followed guidelines outlined in a previous study [14] which used TMB as a biomarker to predict the effectiveness of Ipilimumab and Tremelimumab in patients with advanced melanoma [16]. Recent research has demonstrated that patients with high TMB levels have a greater response rate to PD-1/PD-L1 immune checkpoint inhibitors when compared to those with low TMB levels. Therefore, our analysis included a comparison of TMB levels between CPI-positive and CPI-negative groups to assess the relative efficacy of immune checkpoint inhibitors in the two groups.

### 2.9 Analysis of the correlation between CuRGs as well as CPI and immune cell infiltration

We used single-sample gene set enrichment analysis (ssGSEA) to evaluate the level of immune infiltration in colorectal cancer, and then evaluated the correlation between CuRGs as well as CPI and immune cells through Pearson correlation analysis. Then, we visualized the correlation between CuRGs as well as CPI and 24 types of infiltrating immune cells using the R package ggplot2 and visualized the correlation between immune cell subtypes using the corrplot package. We focused on studying tumor-infiltrating lymphocytes (TILs) including: regulatory T cells (Tregs), T helper (Th) cells, CD8^+^ T cells, T helper 1 (Th1) cells, T helper 2 (Th2) cells, T helper 17 (Th17) cells, natural killer (NK) cells, macrophages, follicular helper T (Tfh) cells, plasmacytoid dendritic cells (pDCs), dendritic cells (DCs), activated dendritic cells (aDCs), immature dendritic cells (iDCs), B cells, mast cells, neutrophils, cytotoxic cells, eosinophils, NK CD56^bright^ cells, NK CD56^dim^ cells, central memory T cells (Tcm), effector memory cells (Tem), and γδ T cells (Tgd). We performed a Pearson correlation analysis to examine the correlation between cuproptosis and immune microenvironments by measuring the correlation between CPI and immune cell fractions.

### 2.10 Enrichment analysis of CuRGs in 20 types of cancer using GSEA

Based on the results of the median of CPI, the cancer samples were divided into a high CPI group and a low CPI group. The first step involved selecting the top 30% of patients from each group for differential analysis. The results were then arranged in descending order of logFC. Next, a GSEA(Gene Set Enrichment Analysis) analysis was conducted on the hallmarks of cancer to determine their relationship with CPI. Finally, this relationship was visualized through a heatmap plot.

### 2.11 Ethics approval and consent to participate

This research does not involve human subjects, animal experiments, or data collection and does not touch upon potentially ethical risks or sensitive areas. Therefore, ethical review is not required for this study. All authors have read and adhered to the relevant ethical and moral guidelines, ensuring that the research conforms to academic and industry standards.

## 3 Results

### 3.1 The gene aberrant expression level of CuRGs in pan-cancer

**Fig 1:**
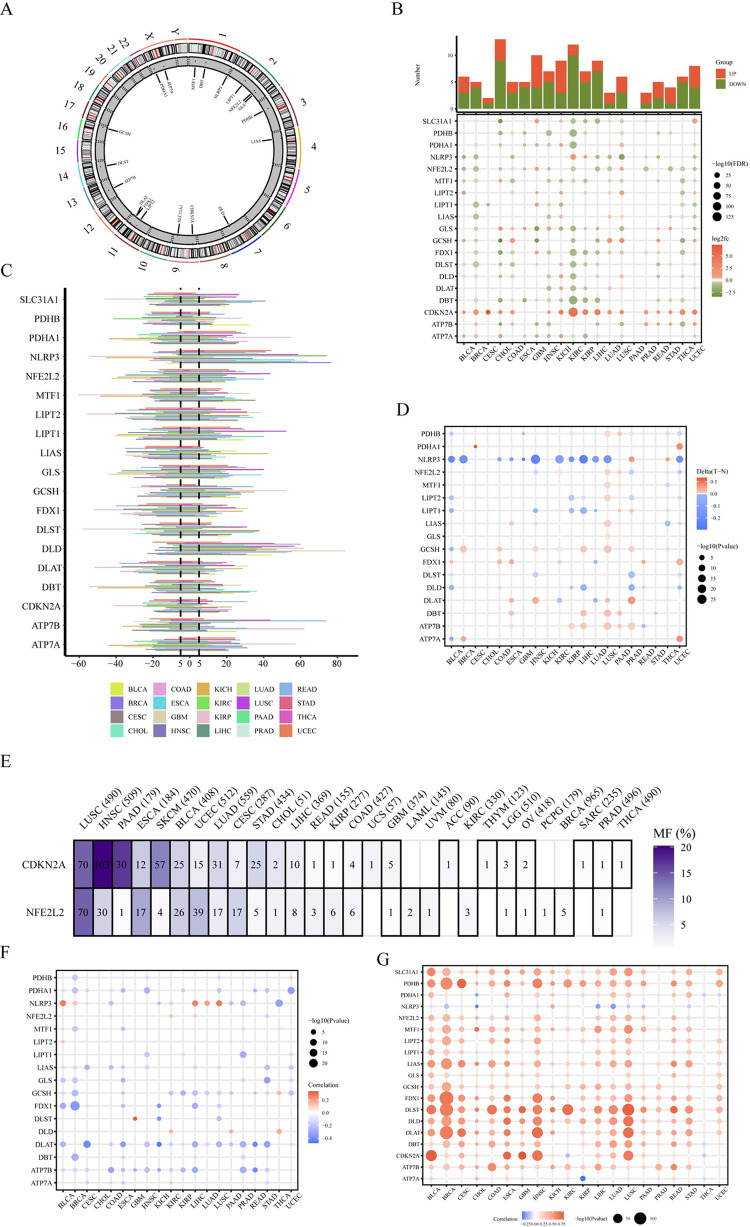
The genetic characteristics and variations of cuproptosis related genes. (A) The location of cuproptosis related genes (CuRGs) on chromosomes. (B) The bar chart above the heatmap shows the number of significantly differentially expressed genes in each cancer type, and the heatmap displays the fold change and FDR of CuRGs in each cancer type. Significant upregulated and downregulated genes (FDR < 0.05) are marked in red and green, respectively. (C) The bar chart shows the frequency of somatic copy number alterations (SCNAs) of each CRG in each cancer type (amplification is positive, deletion is negative). (D) The heatmap shows the differential methylation levels of CuRGs in tumor and normal samples in each cancer type using Wilcoxon rank-sum test. Significantly hypermethylated and hypomethylated genes are marked in red and blue, respectively (*P* < 0.05). (E) The heatmap shows the mutation frequency of CuRGs in various cancers. (F) The correlation between SCNAs and gene expression of CuRGs (Spearman correlation analysis, *P* < 0.05, positively correlated in red, negatively correlated in blue). (G) The correlation between methylation and gene expression of CuRGs (Spearman correlation analysis, *P*< 0.05, positively correlated in red, negatively correlated in blue).

Building upon the foundational studies, particularly the work of Tsvetkov et al [1], we have further explored the 19 genes they identified, known as CuRGs (Cuprotosis Related Genes), to understand their roles in the development and occurrence of cuprotosis. These genes include NFE2L2 (NFE2 Like BZIP Transcription Factor 2), NLRP3 (NLR Family Pyrin Domain Containing 3), ATP7B (ATPase Copper Transporting Beta), ATP7A (ATPase Copper Transporting Alpha), SLC31A1 (Solute Carrier Family 31 Member 1), FDX1 (Ferredoxin 1), LIAS (Lipoic Acid Synthetase), LIPT1 (Lipoyltransferase 1), LIPT2 (Lipoyl(Octanoyl) Transferase 2), DLD (Dihydrolipoamide Dehydrogenase), DLAT (Dihydrolipoamide S-Acetyltransferase), PDHA1 (Pyruvate Dehydrogenase E1 Subunit Alpha 1), PDHB (Pyruvate Dehydrogenase E1 Subunit Beta), MTF1 (Metal Regulatory Transcription Factor 1), GLS (Glutaminase), CDKN2A (Cyclin Dependent Kinase Inhibitor 2A), DBT (Dihydrolipoamide Branched Chain Transacylase E2), GCSH (Glycine Cleavage System Protein H), and DLST (Dihydrolipoamide S-Succinyltransferase).The positions of CuRGs on the chromosome are shown in Fig 1A, where the necessary genes for cuproptosis, LIAS, DLD, DLAT, FDX1, and LIPT1, are located on autosomes, and the cuproptosis inhibitory gene ATP7A is located on the X chromosome. To identify the dysfunctional patterns of 19 CuRGs across various types of cancer, we analyzed mRNA expression profiles, SCNA, and DNA methylation data from both tumor and normal tissues in 20 different cancer types.

We conducted a differential expression analysis to compare the gene expression patterns of CuRGs between tumor and adjacent normal tissues for each of the cancer types under investigation. The primary objective of this analysis was to identify any significant alterations in gene expression patterns. The results of the differential expression analysis are shown in Fig 1B. Our findings indicate that all of the CuRGs analyzed displayed differences in expression levels in one or more types of cancer. Moreover, cross-cancer analysis revealed consistent expression patterns for several CuRGs. CDKN2A is highly expressed in almost all cancer types, while MTF1 is lowly. LIAS is a gene that, when mutated, causes the stabilization of HIF1α in a non-hydroxylated state [17] and exhibits high levels of expression in KICH (Please refer to Table 1 for the abbreviations of cancer names.), LUAD, and LUSC, while showing relatively low levels of expression in BRCA, HNSC, STAD, THCA, and UCEC. DLAT DLAT participates in the metabolism of pyruvate and glycolysis pathways (BioCyc) which is highly expressed in KICH, LUAD, LUSC, and UCEC, while it is lowly expressed in BRCA, HNSC, KIRC and KIRP. FDX1 may affect the prognosis and regulate the metabolism of lung adenocarcinoma [18] and is highly expressed in GBM and UCEC but lowly expressed in 12 other types of cancer. The difference between CHOL and THCA is relatively large. The cuproptosis inhibitory genes, ATP7B and ATP7A, as well as the remaining cuproptosis promoting CuRGs, are highly heterogeneous in tumors but tend to be lowly expressed, which may suggest a lower activation level of cuproptosis in cancer patients.

### 3.2 The genetic alteration level of CuRGs in pan-cancer

To investigate whether SCNA is also related to the occurrence of cuproptosis in cancer, we calculated the percentage of SCNA in CuRGs in pan-cancer. High-frequency copy number gains occurred when SCNA frequencies were greater than 5%, and high-frequency copy number losses occurred when SCNA frequencies were less than 5%. The results in Fig 1C show that except for THCA, all other cancers had high-frequency SCNAs. Among these cancers, the cuproptosis key genes, LIAS, FDX1, and DLAT tended to show copy number deletion, while DLD tended to show copy number amplification. Among the cuproptosis inhibitory genes, CDKN2A, ATP7B, and MTF1 tended to have copy number deletion, while GLS tended to have copy number amplification.

### 3.3 The gene methylation level of CuRGs in pan-cancer

It has been reported that promoter methylation also plays an important role in the development of cancer [19]. The results in Fig 1D show that the methylation levels of CuRGs differ in different cancers. The cuproptosis key genes, LIPT1, and DLD tend to have low methylation, while LIAS, FDX1, and DLAT tend to have high methylation. Similarly, the cuproptosis inhibitory genes, GLS, MTF1, and ATP7B, tend to have high methylation. Surprisingly, there was no difference in CDKN2A expression between normal and tumor groups, indicating that CDKN2A may play a crucial role in preventing cuproptosis, unlike GLS, MTF1, and ATP7B.

### 3.4 The expression profile of CuRGs in relation to genetic alteration and methylation of CuRGs analysis

We assessed the mutation frequency of CuRGs across various cancer types. Fig 1E reveals that HNSC, LUSC, and PAAD have the highest occurrences of CDKN2A mutations. Overall, our multi-omics analysis suggests that pan-cancer dysregulation of CuRGs may be associated with SCNA and DNA methylation. To further verify this hypothesis, we analyzed the relationship between CuRGs expression profile and SCNA and DNA methylation. Firstly, we examined the Pearson correlation between gene expression and copy number segments from TCGA with masked copy numbers. The results showed that the expression profiles of almost all CuRGs were positively correlated with copy number variation (Fig 1F), indicating that copy number variation of CuRGs may affect their expression levels. In addition, Aside from SCNA, promoter methylation can regulate gene expression, and abnormal DNA methylation of promoters is associated with tumor development. Although CuRGs exhibit complex methylation patterns in 20 types of cancer, the expression levels of most CuRGs were negatively correlated with their methylation levels (Fig 1G), but DLD showed positive correlation in three types of cancers, KIRC, PAAD, and THCA, suggesting that methylation directly or indirectly promotes the expression of DLD, which is a key gene in cuproptosis. This indicates that methylation has a complex relationship with cuproptosis.

Therefore, all CuRGs exhibit different patterns of regulation by SCNA and methylation in different cancers. This indicates that the expression regulatory patterns of all CuRGs are tumor specific.

### 3.5 Constructing a Cuproptosis Index (CPI) model in pan-cancer

**Fig 2:**
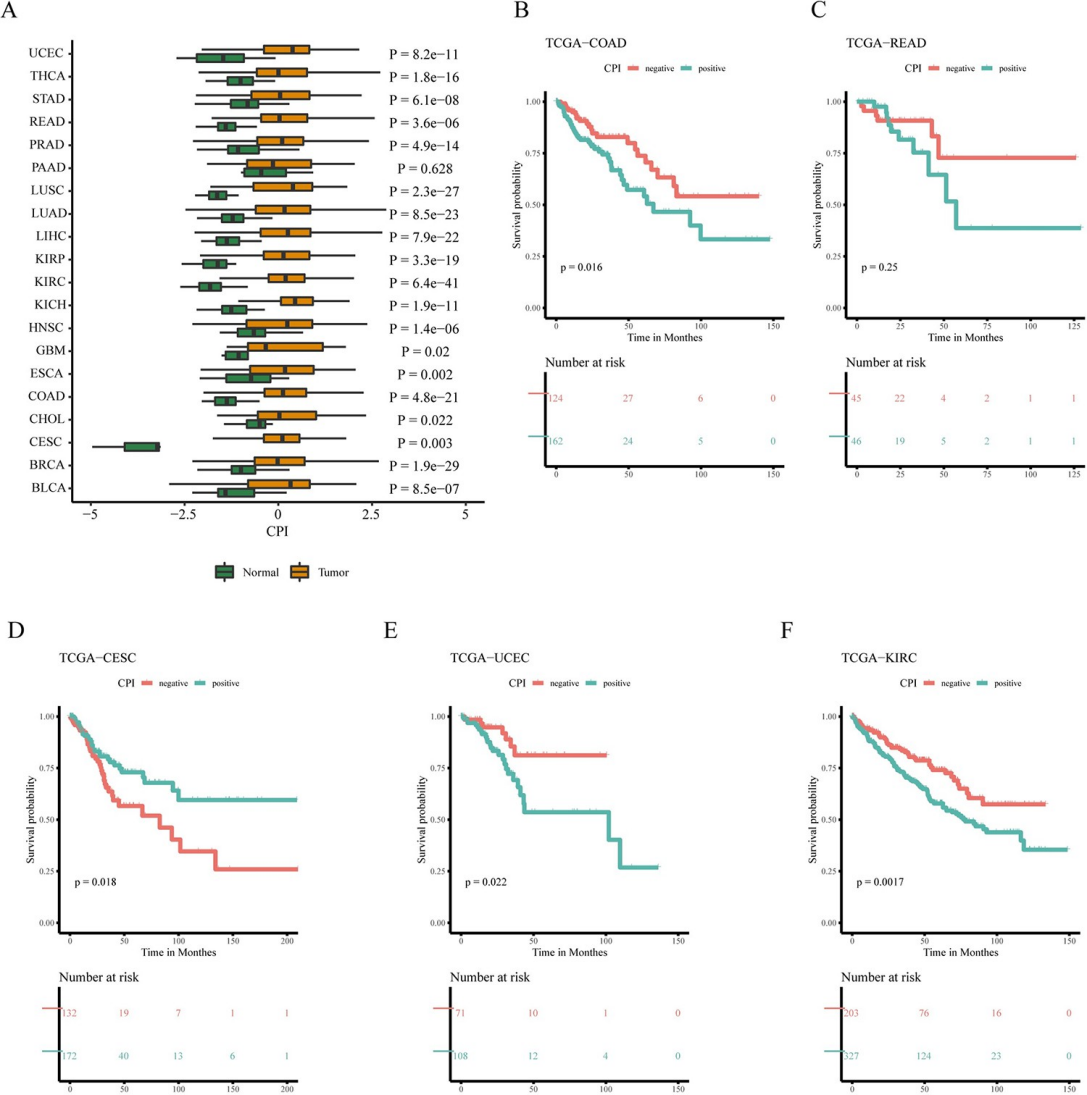
The role of the cuproptosis index (CPI) model in overall cancer prognosis. (A) The difference in CPI between tumor and normal groups in overall cancer. (B-F) Kaplan-Meier survival analysis based on positive and negative groups in CESC, COAD, KIRC, UCEC, and READ.

To comprehensively consider the role of CuRGs in tumor development and evaluate the impact of cuproptosis on patient survival, a CPI model was constructed based on the enrichment score (ES) calculated by subtracting the enrichment score of the negative component from that of the positive component using ssGSEA. The Mann-Whitney U test was used to analyze whether there were differences in CPI between tumor and normal groups. The results in Fig 2A show that, except for PAAD, the CPI of the tumor group in the remaining 19 types of cancers was significantly higher than that of the normal group. According to the positive and negative values of the CPI index, the patients in the tumor group were divided into positive and negative groups. Survival analysis was performed on each cancer type, and the results in Fig 2B–2E show that CESC, COAD, KIRC and UCEC had significant differences, where the survival time of patients in the positive group was significantly lower than that of patients in the negative group. For the other 15 cancer types studied, no significant differences in survival outcomes were observed, as detailed in a S1 Fig. However, unlike in other cancer types studied, the CPI may not be a reliable indicator of prognosis or disease progression in rectum adenocarcinoma patients. This lack of significant association in the READ cohort highlights the potential variability in the utility of the CPI across different types of colorectal cancers.

### 3.6 Independent prognostic analysis of CPI in CRC

**Fig 3:**
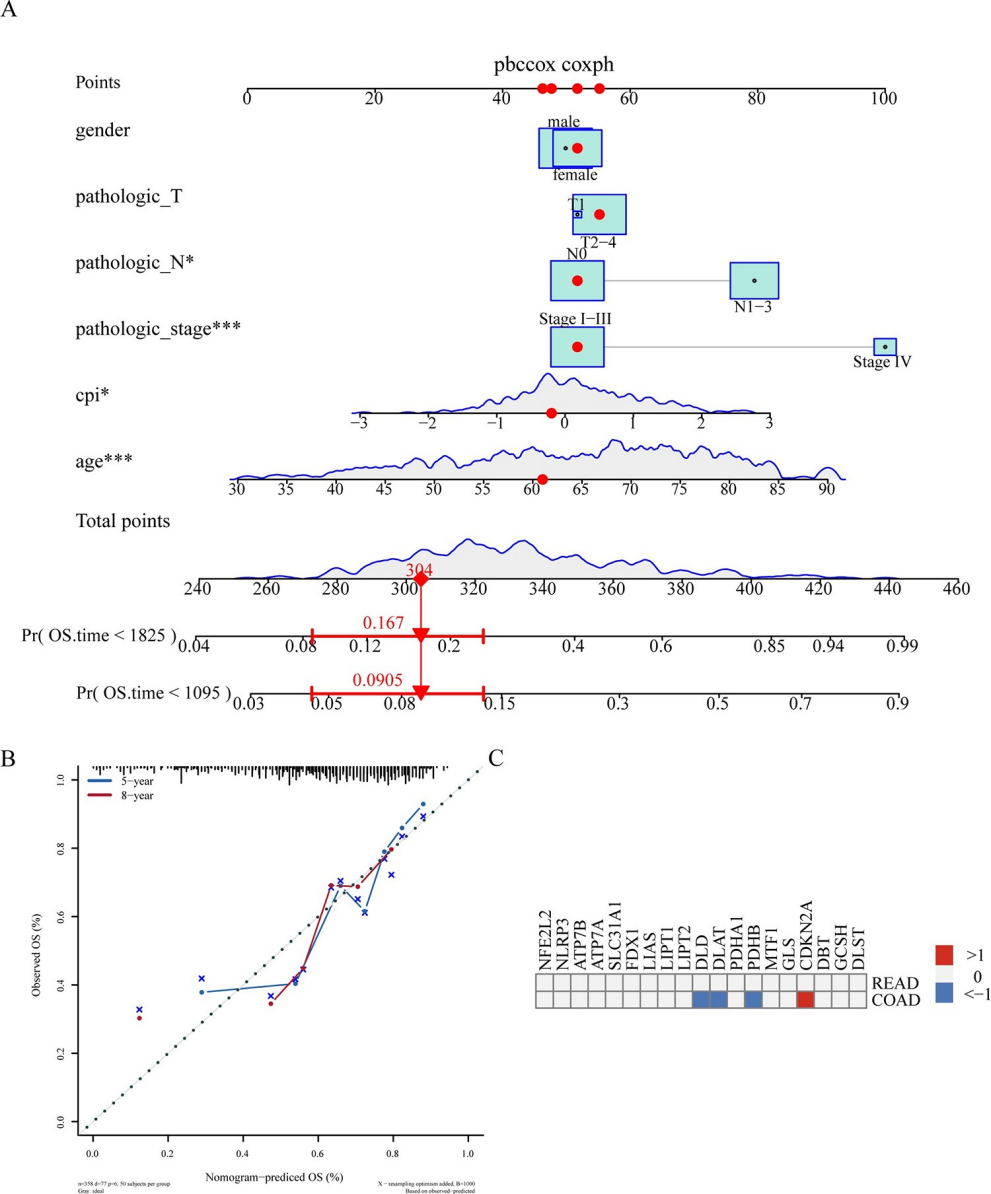
Independent prognostic analysis of CPI in colorectal cancer (CRC). (A) The nomogram shows that CPI can serve as an independent prognostic factor reflecting the prognosis of CRC patients. (B) The calibration plot evaluates the independent prognostic analysis model based on multivariate Cox regression. (C) The heatmap shows the relationship between individual CuRGs in overall cancer and survival through univariate Cox regression. HR > 1 indicates high risk, HR < 1 indicates low risk, and they are marked in red and blue, respectively. Gray is used to indicate where *P*>0.05, which is not significant.

We further explore the influence of the CPI on the prognosis of colorectal cancer (CRC) patients. Utilizing a multivariate Cox regression model, as depicted in Fig 3A, our analysis robustly establishes CPI as an independent prognostic factor in CRC. This model distinctly demonstrates CPI’s predictive significance, which remains consistent even when adjusted for other clinical variables, such as age, gender, and pathological grades and stages. Additionally, the calibration plots presented in Fig 3B are instrumental in assessing the predictive accuracy of our model over a 5-year and 8-year period. These plots, by comparing the predicted and observed survival outcomes, effectively validate the model’s prognostic reliability.

We delve into the analysis of the CuRGs, a univariate Cox regression model was established, to assess its impact on the survival of CRC patients. Our findings in COAD indicate that genes promoting cuprotosis, such as DLD, DLAT, and PDHB, act as protective factors, while CDKN2A, a gene inhibiting cuprotosis, emerges as a risk factor. This observation is in line with the results obtained from our CPI model, demons trating a consistent pattern and underscoring the model’s robustness. Furthermore, in READ, no single gene was found to significantly affect patient survival, aligning with the conclusions drawn from the CPI model. This suggests substantial variability in cuprotosis among CRC subtypes and implies that specific pathways or molecular characteristics in READ might either influence the progression of the disease or mask the features of cuprotosis. These findings call for further research to explore the differential roles of cuprotosis in CRC subtypes and to understand the underlying mechanisms that could explain the distinct impact on COAD and READ.

### 3.7 Genomic feature analysis of CPI-based in CRC

**Fig 4:**
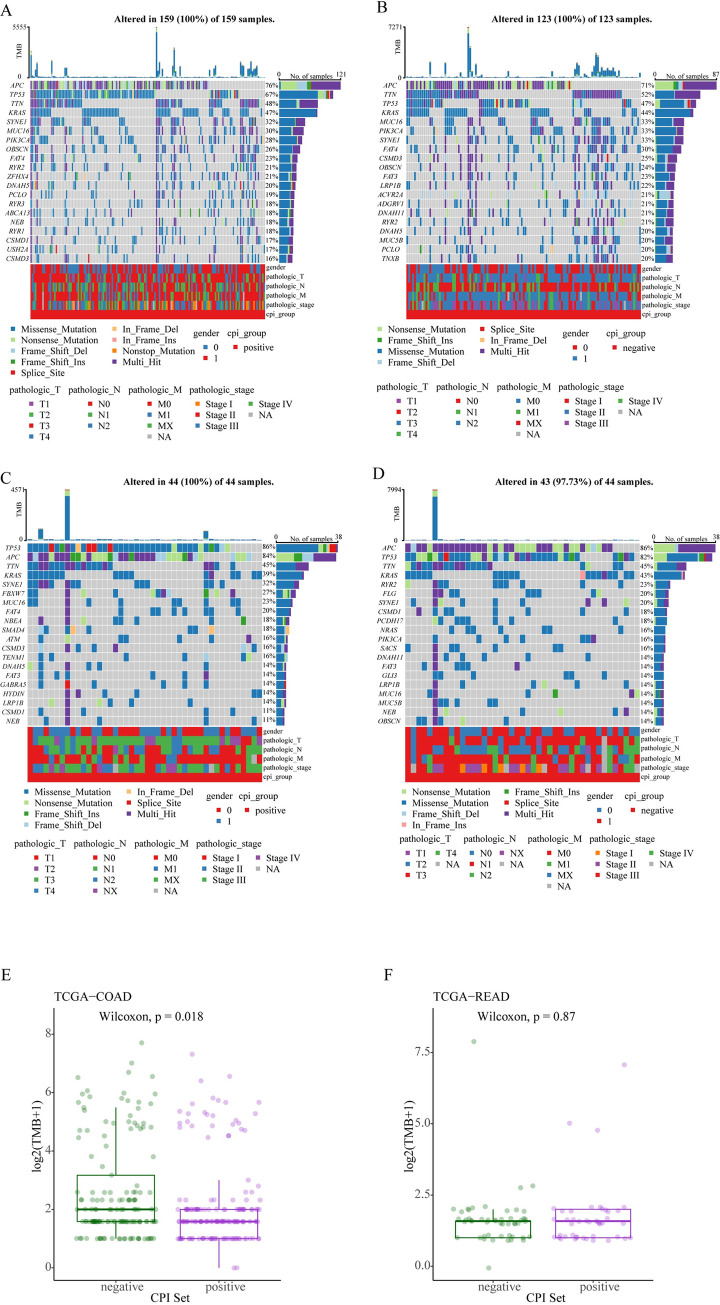
Genomic features based on CPI in CRC. (A) The waterfall plot displays the gene mutation profile in the CPI positive group of COAD (colon adenocarcinoma). (B) The waterfall plot illustrates the gene mutation profile in the CPI negative group of COAD. (C) The waterfall plot shows the gene mutation profile in the CPI positive group of READ (rectum adenocarcinoma). (D) The waterfall plot depicts the gene mutation profile in the CPI negative group of READ. In these waterfall plots, the middle bar graph represents the types of gene mutations in each sample, the upper histogram indicates the tumor mutation burden (TMB) for each sample, and the right-side stacked bar graph shows the mutation frequency and type of the respective genes in that group. The lower bar graph provides clinical information about the samples, including gender, tumor staging, and more. (E-F) Through Wilcoxon rank-sum testing, we analyzed the differences in tumor mutation burden (TMB) between the CPI positive and negative groups in both COAD and READ.

Drawing waterfall plot to analyze the mutation spectrum of positive and negative components divided according to the CPI index, the results (Fig 4A–4D) showed that in COAD and READ, the genes with highest mutation rates in different groups were APC, TP53, TTN, and KRAS. The frequency of gene mutations in the positive group in COAD was higher than in the negative group, for example, the mutation rate of APC in the positive group was 76%, while in the negative group it was 84%. However, in READ the opposite was observed, such as the mutation rate of APC being 76% in the positive group and 86% in the negative group. This may suggest that the role of cuproptosis genes in the COAD and READ subtypes of colon cancer is not entirely consistent. We utilized the Wilcoxon rank-sum test to analyze the differences in tumor mutation burden (TMB) between the CPI positive and negative groups in both COAD and READ. This analysis revealed that in COAD, the TMB is higher in the CPI negative group compared to the CPI positive group. However, in READ, no significant differences in TMB were observed between the CPI positive and negative groups. This finding suggests that in COAD, the CPI negative group may have a better response to immunotherapy. It can be hypothesized that cuprotosis might influence patient survival through its interaction with the immune microenvironment.

### 3.8 Analysis of immune infiltration related to CRC

**Fig 5:**
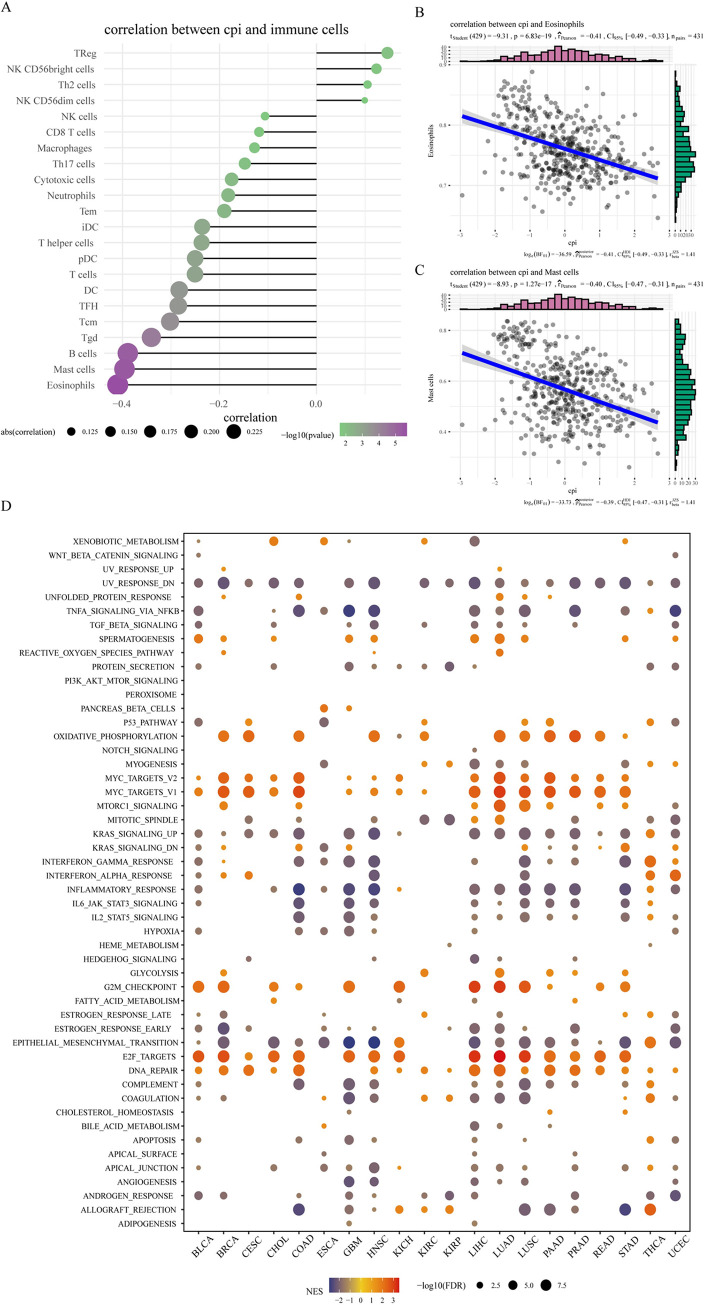
CPI immune infiltration and enrichment analysis. (A-C) Correlation analysis between CPI and immune cells. *P* < 0.05 is considered to indicate a correlation between the two. (D) The heatmap shows we employed the GSEA function within the R package clusterProfiler to assess the relationship of the CPI with various tumor hallmark pathways. This comprehensive analysis resulted in a heatmap that visually represents the enrichment scores of CPI across distinct hallmark pathways, illustrating the multifaceted role of CPI in cancer biology.

By GSVA (Gene Set Variation Analysis) analysis, tumor samples were scored for immune infiltration to quantitatively study immune cells infiltrating different types of tumors. Pearson correlation analysis was used to explore the relationship between immune infiltration and CuRGs and CPI. The results showed (Figs 5A and S2) that the degree of immune infiltration of TReg, NK CD56^bright^ cells, Th2 cells, and NK CD56^dim^ cells was positively correlated with the CPI, while the degree of immune infiltration of Tem, Tgd, B cells, Mast cells, Eosinophils, and other cells was negatively correlated. In addition, cuproptosis key genes FDX1 and LIAS were negatively correlated with all types of immune cells including TReg, while DLD was positively correlated with Th17 cells and negatively correlated with NK cells. DLAT was positively correlated with Th2 cells and negatively correlated with NK cells, and LIPT1 was negatively correlated with Neutrophils. These results suggest that cuproptosis may play a role in depleting immune cells. To provide a more detailed visualization of these relationships, scatter plots depicting the negative correlations between CPI and Eosinophils as well as Mast cells have been added (Fig 5B and 5C). These plots further reinforce the observed trends. For a comprehensive view of the correlations between CPI and a broader range of immune cells, readers are directed to S3 Fig. These results collectively suggest that cuprotosis may have a significant role in modulating immune cell presence in the tumor microenvironment, indicating its potential impact on tumor immunity and patient prognosis."

### 3.9 GSEA enrichment analysis based on CPI

Using the GSEA function in the R package clusterProfiler, the enrichment scores of CPI in various pathways of tumor hallmarks were calculated and presented in the form of a heatmap. The results showed (Fig 5D) that CPI was significantly associated with 48 hallmark pathways. For example, high CPI (positive group) was enriched in pathways such as Oxidative Phosphorylation, Myc Targets V2, Myc Targets V1, mTORC1 Signaling, G2M Checkpoint, E2F Targets, indicating that cuproptosis promotes cancer through the above carcinogenic pathways. In addition, low CPI (negative group) was significantly enriched in pathways such as UV Response DN, TNFA Signaling via Nfkb, Inflammatory Response, suggesting that cuproptosis related genes inhibit cancer development through the above pathways. These contrasting patterns of pathway enrichment in relation to high and low CPI scores provide insights into how cuprotosis, as quantified by the CPI, may either promote or inhibit cancer depending on the specific molecular pathways involved. The heatmap in Fig 5D serves as a crucial tool for visualizing these complex associations, offering a clearer understanding of CPI’s role in the intricate network of cancer hallmark pathways.

## 4 Discussion

Copper is an essential element in the human body, and its redox characteristics are a "double-edged sword" for cells. Copper ions are involved in many cellular processes including mitochondrial respiration, antioxidant defense, redox signaling, kinase signaling, and autophagy [20]. Recent studies have found that copper ions also mediate a novel form of cell death called cuproptosis, and research has shown that copper directly binds to acylated components of the TCA cycle and subsequent Fe-S cluster protein loss triggers cell death [1,21]. FDX1, a key gene in cuproptosis encodes a reductase that reduces Cu^2+^ to Cu^+^, facilitating DLAT lipation and is a direct target of elesclomol. In addition, FDX1 also increases the level of reactive oxygen species (ROS) within cells, ultimately leading to cell death [22].

Our results elucidate the multi-omics characteristics of cuproptosis related genes in pan-cancer, establish a CPI index model that predicts the prognosis of CRC patients and evaluates the correlation between CuRGs and immune infiltration levels. In recent cuproptosis related studies, Huang et al. proposed a risk model for cuproptosis related lncRNAs to predict the prognosis of hepatocellular carcinoma patients [23]. Xu et al. developed a strategy to induce copper poisoning using copper-based nanomaterials to treat cancer [24]. However, a systematic description of CuRGs in pan-cancer cohorts has not yet been conducted, including the overall changes in gene expression levels, epigenetic and genetic variation levels in the TCGA pan-cancer cohort. Therefore, we conducted a pan-cancer analysis of 20 cancers based on cuproptosis related genes and revealed the global changes in CuRGs at the genetic, expression, and epigenetic levels. We also established a CPI index based on ssGSEA algorithm and expression profile data to measure the level of cuproptosis. Subsequently, through survival analysis, we found that CPI could effectively distinguish the prognosis of CESC, COAD, KIRC and UCEC. We then selected CRC, which we were interested in, to further explore the correlation between CPI and CRC patients’ survival prognosis, genetic factors, tumor mutation burden, and immune infiltration.

The way in which CuRGs regulate tumor behavior is not clear. In this study, the gene expression of the vast majority of CuRGs was downregulated in most cancers, except for CDKN2A, which was upregulated. Studies have shown that CDKN2A may promote the metastasis of colorectal cancer cells by inducing epithelial-mesenchymal transition (EMT) [25]. At the same time, Fig 1C shows that the SCNA of CDKN2A was significantly amplified in both COAD and READ. Similarly, CDKN2A is also one of the most common genetic changes in pancreatic cancer precursors, mainly manifested as deletion [26]. Compared to the normal group, FDX1, a key gene of cuproptosis in the tumor group, was highly methylated, and FDX1 provides electrons to initiate a free radical chain reaction catalyzed by acyl synthetase. Lipation is also the target of the toxic anti-tumor copper ion carrier elesclomol [22,27]. Methylation of this gene may inhibit the occurrence of cuproptosis. Overall, the genetic changes of CuRGs affect the occurrence and development of cancer by regulating cuproptosis.

The CPI index constructed based on the expression profile of CuRGs can effectively measure the level of cuproptosis in pan-cancer. The results showed that in CRC, patients in the positive group had significantly lower prognosis than those in the negative group. CPI can also serve as an independent prognostic factor and compared to the impact of individual CuRGs on patient prognosis (CDKN2A as a risk factor, PDHB, DLAT, DLD as protective factors), CPI better reflects the comprehensive impact of CuRGs on patient prognosis. We also found that the mutation spectrum analysis in COAD and READ showed that the role of cuproptosis genes in the COAD and READ subtypes of CRC was not entirely consistent. Furthermore, in the immune infiltration analysis, the expression of cuproptosis key genes FDX1 and LIAS was negatively correlated with all types of immune cell infiltration, including TReg, indicating that cuproptosis may interact with the immune system to jointly regulate the occurrence and progression of cancer.

High CPI is significantly enriched in the pathways Myc Targets V1, Myc Targets V2, mTORC1 Signaling, G2M Checkpoint, and E2F Targets. According to previous studies, some compounds that directly or indirectly inhibit MYC have demonstrated anti-cancer activity in preclinical tumor models [28]. As a protein kinase, mTOR is influenced by nutrient levels and growth signals, and its mediated metabolic reprogramming plays an important role in stem cells and immune cells [29]. This reveals the correlation between cuproptosis and carcinogenic pathways, which suggests how CuRGs promote the development of cancer through certain pathways.

In summary, we analyzed the multi-omics characteristics of cuproptosis-related genes in pan-cancer. We also constructed a CPI model that responds to the level of cuproptosis and investigated the correlation between CPI and clinical prognosis, genetic alterations, and immune infiltration in colon cancer, which is of particular interest. Thus, we have provided new insights into the relationship between cuproptosis and the occurrence as well as prognosis of cancer.

## Supporting information

S1 Fig This figure presents Kaplan-Meier survival analysis curves based on CPI positive and CPI negative groups across 20 different cancer types. (TIF)

S2 Fig Line graphs depicted the correlations between individual Cuprotosis Related Genes (CuRGs) and the CPI with various immune infiltration cells. (TIF)

S3 Fig Scatter plots illustrate the correlations between individual Cuprotosis Related Genes (CuRGs) and the (CPI) with different types of immune infiltration cells. Each scatter plot demonstrates the relationship of a specific CuRG or CPI with an immune cell type. A correlation is considered significant if p < 0.05.
(PDF)

## Abbreviations

CRC: colorectal cancer
CuRGs: cuproptosis related genes
DEGs: differential expression genes
EMT: epithelial-mesenchymal transition
ES: enrichment score
HR: hazard ratio
LCD: lysosome-dependent cell death
logFC: log Fold Change
OS: overall survival
PDH: pyruvate dehydrogenase
SCNAs: somatic copy number alterations
ssGSEA: gene set enrichment analysis
TCGA: the Cancer Genome Atlas
TMB: tumor mutation burden
