# Correction: Systematic analysis of cuproptosis abnormalities and functional significance in cancer

**DOI:** 10.1371/journal.pone.0362635

**Published:** 2026-11-12

**Authors:** Shang Rumin, Xiangming Han, Cui Zeng, Fei Lv, Rong Fang, Rongrong Gong, Xiaochang Tian, Xiangwu Ding

The Data Availability statement in [1] is incorrect. The following information is missing from the Data Availability statement: The minimal dataset underlying the findings of this study are available at https://doi.org/10.5281/zenodo.21245128. The deposited dataset includes the original images, original data, and source data underlying the figures, tables, statistical analyses, and main findings reported in the article.
