# Supplementary figures and images for: Systematic analysis of cuproptosis abnormalities and functional significance in cancer
Source: PLoS One. 2024 Apr 4;19(4):e0300626. doi: 10.1371/journal.pone.0300626 (PMC10994309; doi:10.1371/journal.pone.0300626)

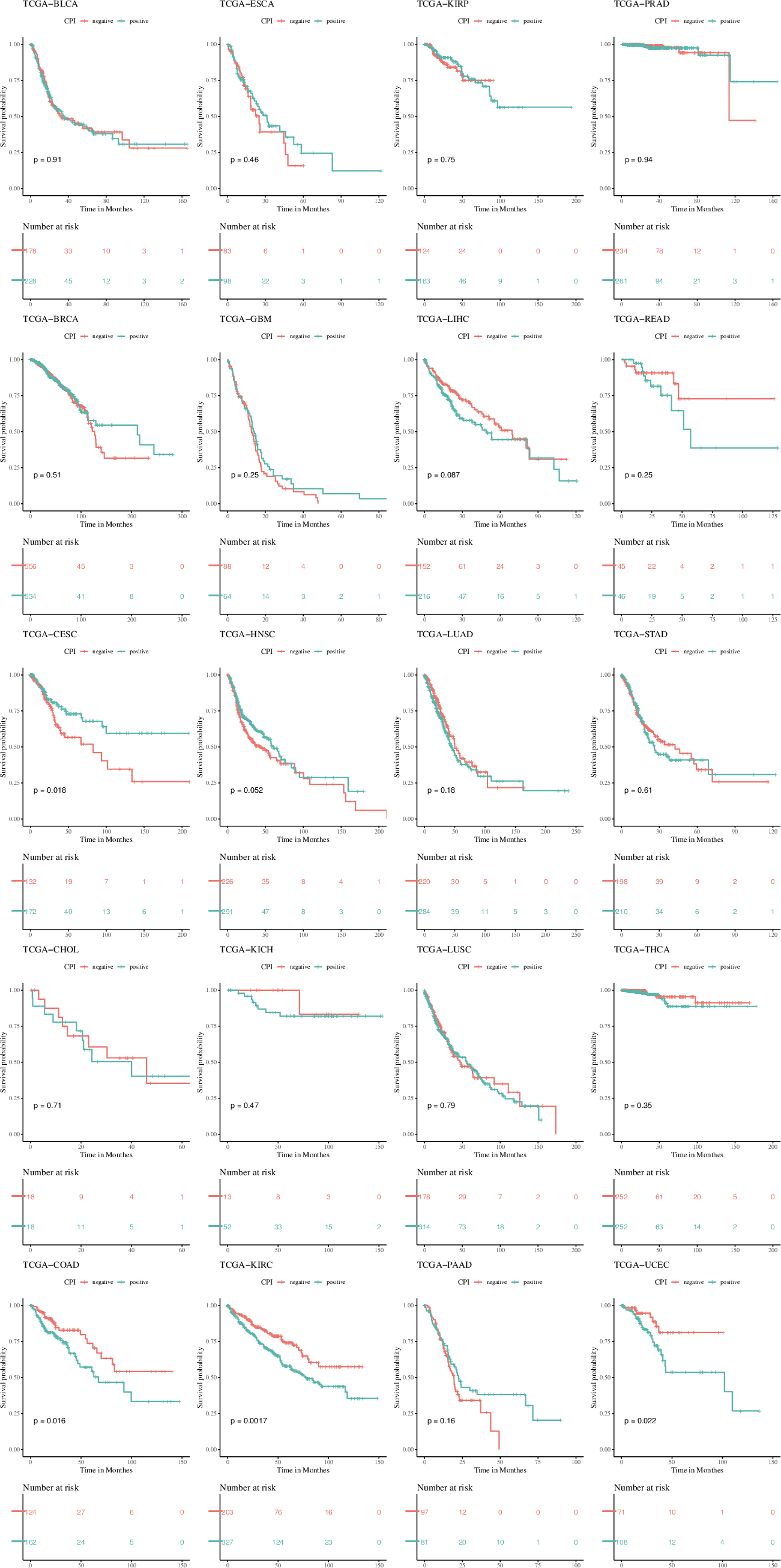

Supplement: S1 Fig — (TIF) [file pone.0300626.s001.tif]

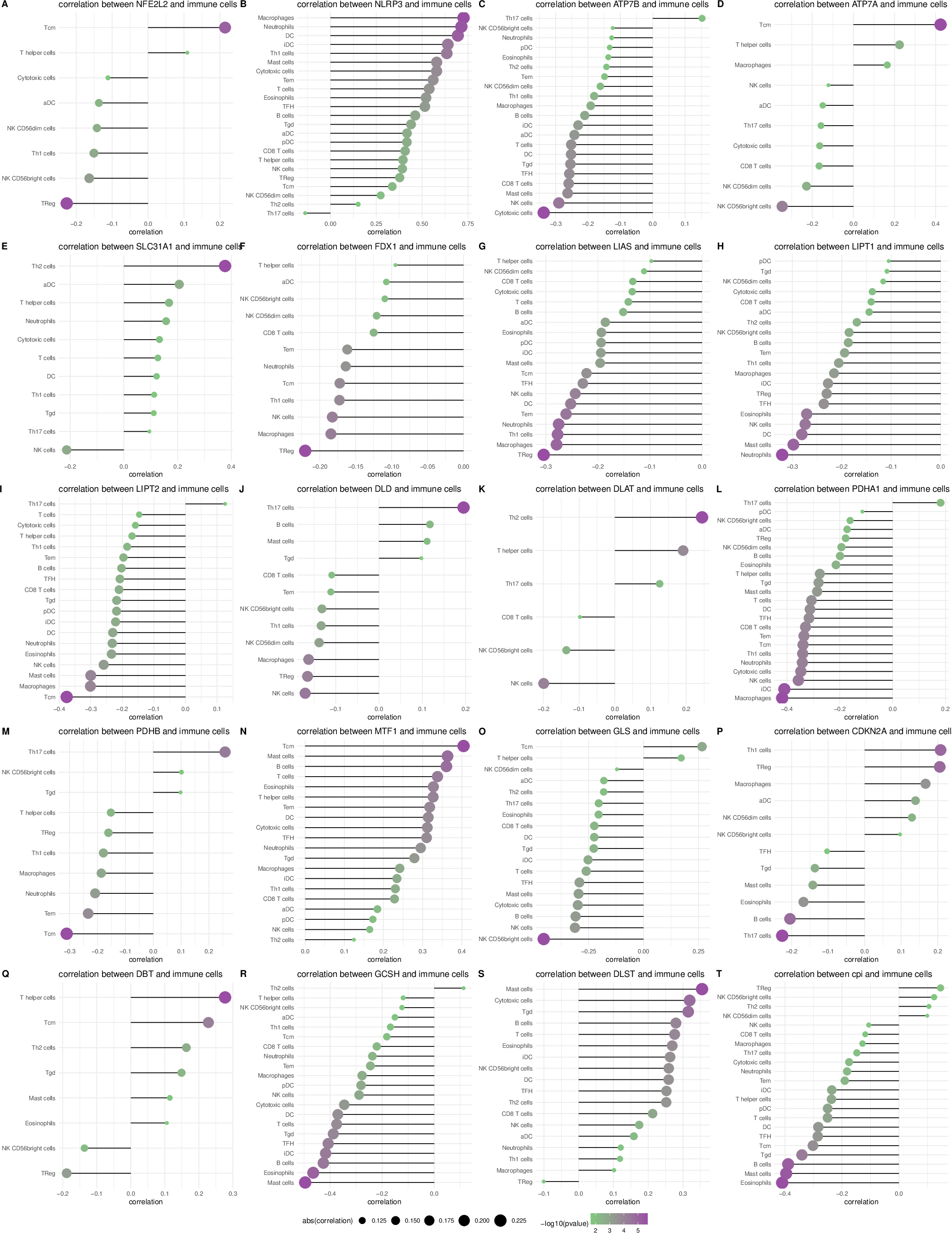

Supplement: S2 Fig — (TIF) [file pone.0300626.s002.tif]

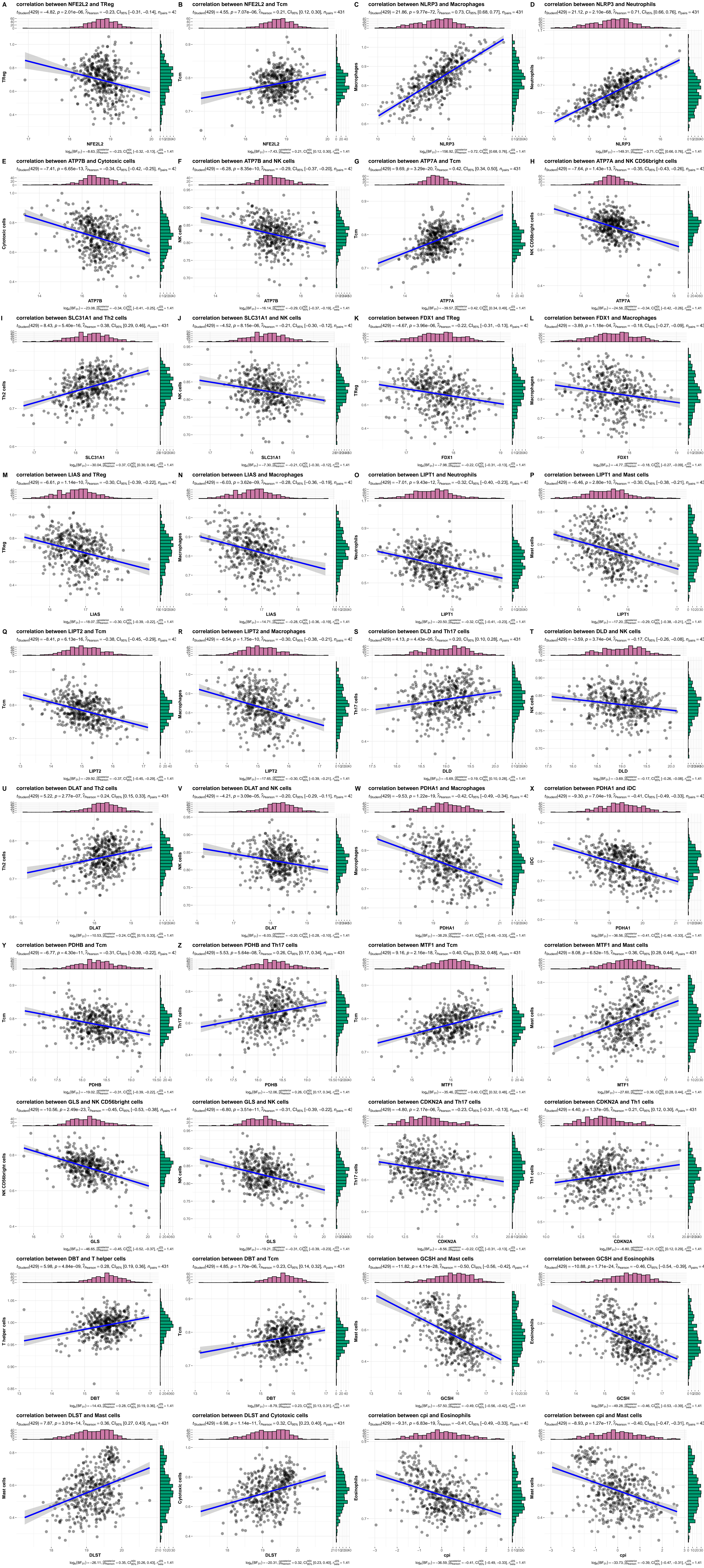

Supplement: S3 Fig — Each scatter plot demonstrates the relationship of a specific CuRG or CPI with an immune cell type. A correlation is considered significant if p < 0.05. (PDF) [file pone.0300626.s003.pdf]
